# Sex-differences in COVID-19 diagnosis, risk factors and disease comorbidities: A large US-based cohort study

**DOI:** 10.3389/fpubh.2022.1029190

**Published:** 2022-11-17

**Authors:** Samer A. Kharroubi, Marwa Diab-El-Harake

**Affiliations:** ^1^Department of Nutrition and Food Sciences, Faculty of Agricultural and Food Sciences, American University of Beirut, Beirut, Lebanon; ^2^School of Health and Related Research, The University of Sheffield, Sheffield, United Kingdom

**Keywords:** sex differences, COVID-19, Healthjump data, SARS-CoV-2, comorbidities

## Abstract

**Introduction:**

Morbidity and mortality from COVID-19 are higher among men, however, underlying pathways remain controversial. We aim to investigate sex-gender differences in COVID-19 in a large US-based cohort, namely COVID-19 Research Database. More specifically, the objectives are to explore the socio-economic characteristics of COVID-19 male and female patients and to examine potential sex differences in lifestyle factors and disease comorbidities among diagnosed patients.

**Methods:**

This is a retrospective cohort study contrasting male vs. female patients with test-confirmed COVID-19. The study used Healthjump electronic medical records (e.g., demographics, encounters, medical history, and vitals) extracted from January 2020 to December 2021 (*N* = 62,310).

**Results:**

Significant sociodemographic and comorbidity differences were observed between males and females (*p* < 0.05). For example, a significantly higher proportion of males (vs. females) were aged ≥70-year-old (17.04 vs. 15.01%) and smokers (11.04 vs. 9.24%, *p* < 0.0001). In addition, multiple logistic regression showed that hypertension and diabetes were significantly more frequent in males [adjusted odds ratio (ORa) = 66.19 and ORa = 22.90].

**Conclusions:**

Understanding the differences in outcomes between male and female patients will inform gender equity responsive approach to COVID-19 and enhance the effectiveness of clinical practice, health policy and interventions.

## Introduction

The novel coronavirus disease 2019 (COVID-19), caused by the severe acute respiratory syndrome coronavirus 2 (SARS-CoV-2), has rapidly accelerated worldwide and, on March 2020, it was declared as a global pandemic by the World Health Organization. As of May 2022, a total of 524,339,768 confirmed COVID-19 cases has been revealed globally including 6,281,260 deaths (1). The United States (US) was mostly hit by the pandemic in terms of number of cases (over 82 million people) and deaths (over 900,000 individuals) (1).

Emerging evidence indicates that male sex is becoming a potential risk factor for COVID-19 death and more severe disease. Nearly all countries with known sex-disaggregated data show a male bias in COVID-19 mortality and the risk of death is almost 1.7 times greater in males than in females (2). Many theoretically grounded hypotheses may explain the potential male bias in Covid-19 outcomes, such as gender-related social factors including gender-linked health behaviors and occupational exposures, that overlap with other socioeconomic factors like employment and race/ethnicity (3).

Recent studies suggest gender disparities in the COVID-19 clinical outcomes, see for example (4–7). Emerging evidence suggests sex-based or gendered differences potentially due to immunological factors (8–10). Some mechanisms underline the influence of hormonal factors (11), expression of the angiotensin converting enzyme 2 (ACE-2) receptors in the lungs (9), smoking (12), among others (12–14). Further evidence shows an early sign of gender-specific patterns in diseases worldwide. As of May 20, 2021 and based on available sex-disaggregated data from the Global Health 50/50 investigators, the infection fatality rate (IFR) in males vs. females showed higher fatality rates in men, and this was also the case in other countries like Brazil, Yemen, Mexico, Scotland, and Guatemala. In total, men had significant higher odds of death from COVID-19 disease in 49 countries, when compared to women (15). As of May 2022, the latest data from the US showed a higher proportion of deceased male patients from COVID-19 vs. females (55% males and 45% females). Researchers has become increasingly concerned about significant sex and gender disparities in the prevalence, incidence and prognosis of patients with COVID-19.

In addition, various research studies showed that males had higher rates of mortality, hospitalization, and clinical complications from COVID-19 compared to females (16–18). For instance, male sex was independently correlated with in-hospital mortality of COVID-19 patients in China (4). Males were shown to have significantly greater rates of hospitalization, ICU transfer, vasopressor support, and endotracheal intubation in a multicenter retrospective cohort study comparing male vs. female COVID-19 patients in the Rush University System, Chicago, USA. Male sex and mortality were also significantly correlated in the entire sample of US patients after controlling for age and illness comorbidities (17).

Using a large US-based cohort, we aim in this research to investigate sex-differences in COVID-19. In particular, the objectives are (16) to describe temporal trends in COVID-19 prevalence over time and to summarize age-and sex-distribution of cases among male and female patients, (1) to explore the demographic and socio-economic characteristics of COVID-19 male and female patients, and (2) to examine potential sex differences in lifestyle behaviors, risk factors and disease comorbidities among diagnosed patients. Findings from the present study could be beneficial for policy decision makers and global health organizations, as it informs them to consider the sex and gender effects of the COVID-19 pandemic, thereby enhancing the effectiveness of clinical practice, health policy and interventions. Sex-disaggregated data will also help clinicians and researchers to consider sex as a biological variable as well as sex-related social and behavioral factors (including risk factors, lifestyle behaviors, disease comorbidities, etc.) when planning medical treatments and interventions.

## Methods

### Study design and data source

This is a retrospective cohort study contrasting male vs. female patients with test-confirmed COVID-19 (polymerase chain reaction [PCR] + as well as IgG/IgM+) from January 2020 to December 2021. The study used Healthjump electronic medical records (EMR) available from the COVID-19 Research Database consortium (https://covid19researchdatabase.org). Data were extricated by SQL using Snowflake (Snowflake Inc., San Mateo, CA, USA) and were also retrieved from all departments in every hospital enrolled, including inpatient and outpatient hospital along with emergency room. The study is also in accordance with relevant guidelines and regulations.

### The Healthjump database

The Healthjump, available through the COVID19 Research Database, extracts the EMR data and contains demographics, appointments, encounters, medications, procedures, allergies, immunizations, labs, provider, social history, and vitals. For the present research, we focus on appointments, encounters, medical history, diagnosis, procedures, immunizations, reason for visit, social history, and vitals, all specifically for COVID-19 care. Here, the Healthjump EMR sample includes data from inpatient physicians, urgent care and emergency room visits including reason for visits, procedures performed, and laboratory test. The patient's date of birth, race, sex, ethnicity, state and the 3-digit zip code of residence were also included in a demographic file.

### Data analysis

Regarding data analysis, the research team selected key variables to assess sociodemographic and lifestyle factors among COVID-19 patients. Data on social history (e.g., education, ethnicity, and language), demographic (e.g., age and sex), appointment, immunization (type of vaccine), vitals (e.g., oxygen saturation, BP etc.) and diagnoses (e.g., hypertension, diabetes etc.) were all extracted from the Healthjump. Data was all exported to the statistical software STATA for conducting data analysis and performing some descriptive statistics. Frequency tables were generated to present data for disease comorbidities, symptoms, and other categorical variables. Chi-square tests were used to assess statistically significant differences in sociodemographic (e.g., age, education, and ethnicity) and lifestyle (smoking, alcohol, and BMI) between males and females. Simple logistic regression was conducted to examine sex-differences in demographic and social characteristics, laboratory parameters, vaccination, comorbidities/risk factors/pre-existing conditions as well as primary reason for visit (ICD10) among diagnosed patients. Variables that were found to be significantly associated by sex were all added to the final logistic model. Multiple logistic regression was conducted to examine sex-differences in socioeconomic characteristics, lifestyle factors and disease comorbidities (e.g., obesity, smoking, and influenza) among diagnosed patients. To summarize results from the logistic regression models, crude odds ratio (ORc) and adjusted odds ratio (ORa) along with their respective 95% confidence intervals (CI) were used. All reported *p*-values were compared at a significance threshold of 5% and were based on two-sided tests.

## Results

**Figure 1:**
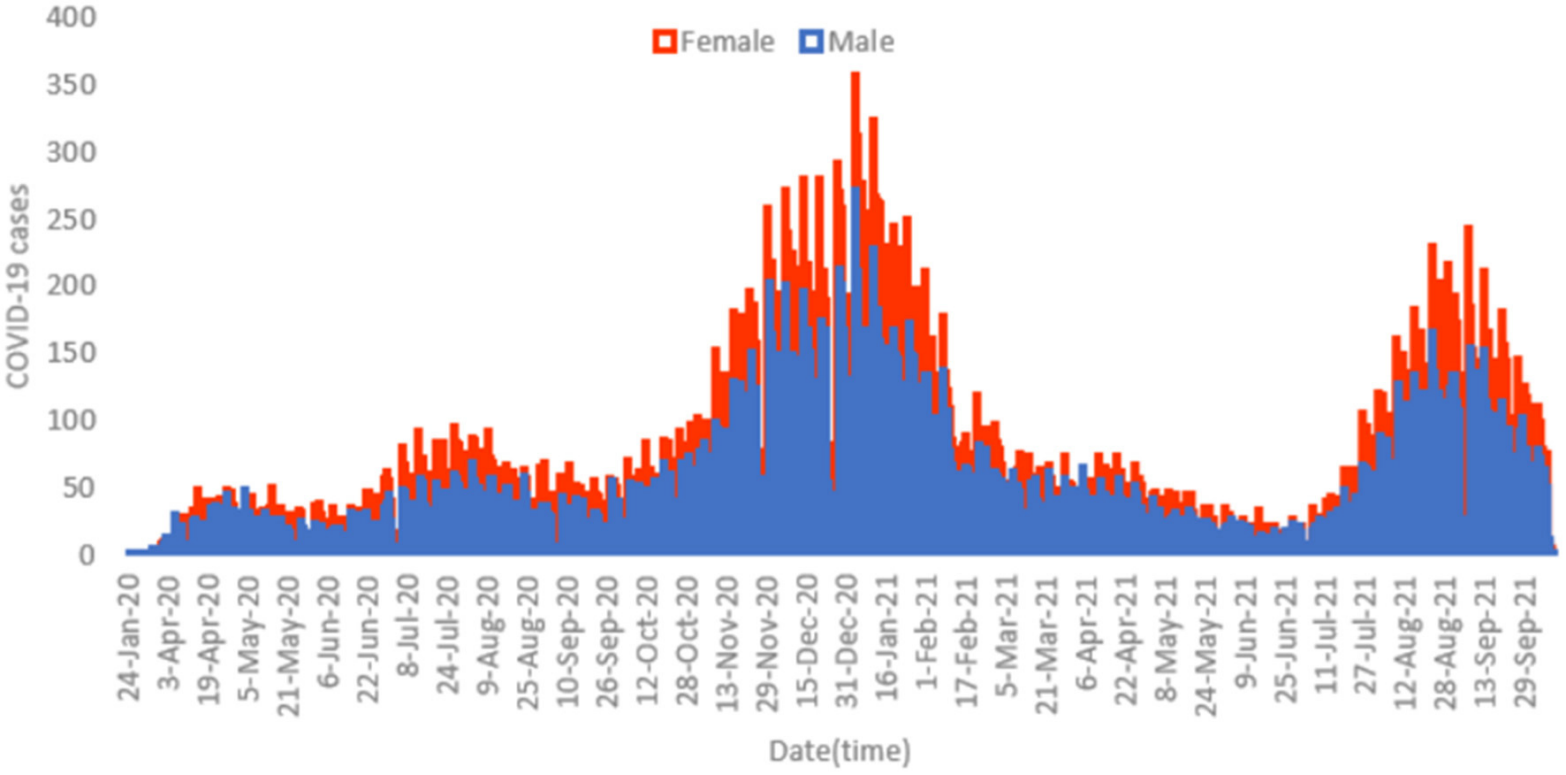
Histogram for daily counts of COVID-19 infections for both male **(blue)** and female **(red)** patients, 2020–2021 (*N* = 62,310).

**Figure 2:**
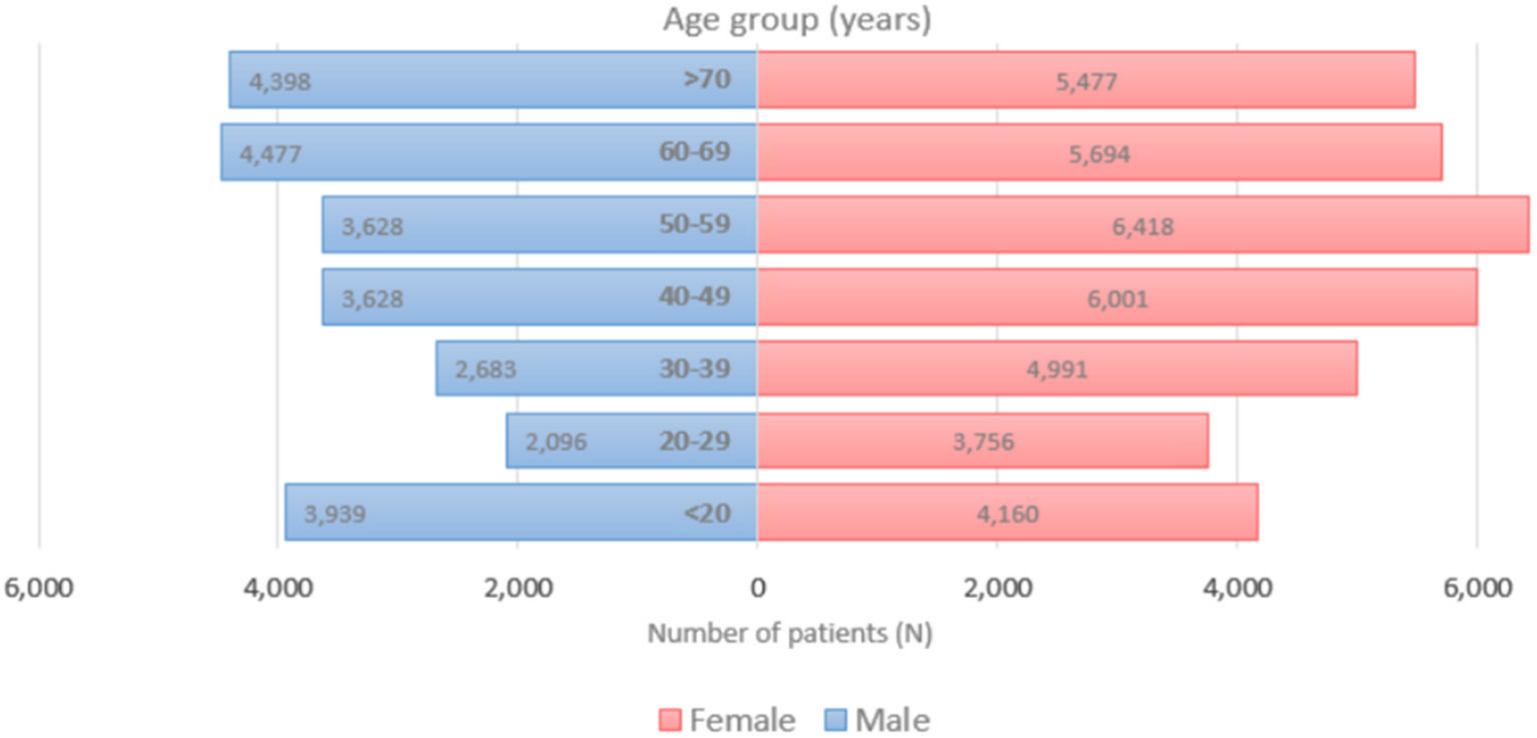
Age distribution of COVID-19 incident cases for both females **(left)** and males **(right)**, 2020–2021 (*N* = 62,310).

Figure 1 displays the overall daily infection counts of COVID-19 cases for both male and female patients within the study period. Figure 1 shows for both genders that the temporal evolution of the daily infection counts of COVID-19 cases has followed an increasing trend and reached a peak at the end of December 2020, which was then followed by a slow decrease and then by a rapid decline until the end of June 2021, where confirmed cases were close to nearly zero levels. After then, the number of diagnosed patients noticeably increase reaching another peak early September 2021. By the end of the study period, the proportion of COVID-19 cases decreased to few cases in December 2021. Figure 2 depicts an increase with age in COVID-19 cases for both male and female patients, affecting the most those with advanced age (50–59 years), with a total of 3,628 male cases and 6,418 female cases.

**Figure 3:**
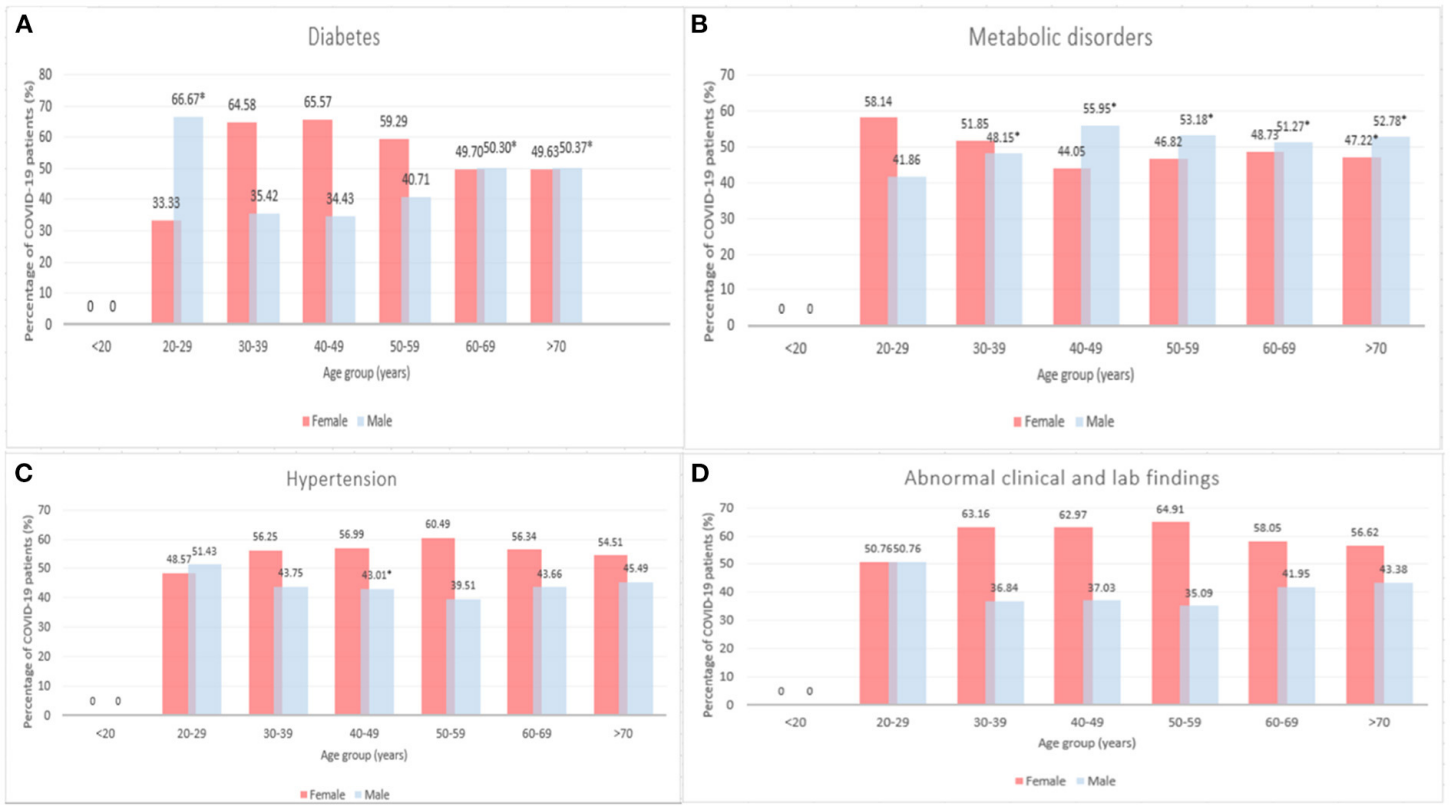
Number of COVID-19 cases among patients with diseases comorbidities by age and sex, 2020–2021 (*p < 0.05). **(A)** Diabetes, **(B)** Metabolic disorders, **(C)** Hypertension, and **(D)** Abnormal clinical and lab findings.

Figure 3 presents the count of COVID-19 cases among patients with diseases comorbidities by age and sex. A significantly higher proportion of males aged 20–29, 60–69, and >70 years had diabetes compared to females in the same age group (66.7 vs. 33.33, 50.30 vs. 49.70, and 50.37 vs. 49.63%, *p* < 0.05, see Figure 3A). A higher percentage of females aged 30–39, 40–49, and 50–59 years had diabetes compared to males, however, this difference was not statistically significant (64.58 vs. 35.42, 65.57 vs. 34.43, and 59.29 vs. 40.71%, *p* > 0.05). There is an age-dependent increase in metabolic disorders among female patients aged 40–49 (44.05%), 50–59 (46.82%), 60–69 years (48.73%) (Figure 3B). On the other hand, the percentage of males with metabolic disorders decreased by age (55.95, 53.18, 51.27% for males aged 40–49, 50–59, and 60–69 years respectively). A significantly higher percentage of males aged 40–49, 50–59, 60–69, and >70 years had metabolic disorder compared to females (55.95 vs. 44.05, 53.18 vs. 46.82, 51.27 vs. 48.73, and 52.78 vs. 47.2215%, *p* < 0.05, see Figure 3B). However, more females aged 30–39 years had metabolic disorders compared to males aged 30–39 years (51.8515 vs. 48.15%, *p* < 0.05). As shown in Figure 3C, significantly more females had hypertension within 40–49 years old age group (56.99 vs. 43.01%). No significant differences were observed in abnormal clinical and lab findings between male and female patients by age groups (*p* > 0.05, see Figure 3D).

Table 1 presents socio-demographic characteristics differences between male and female patients, 2020–2021. A total of 62,310 confirmed COVID-19 patients were included in the analysis of the present study. Overall, 13% of COVID-19 patients in our sample were below 20 years, 9.39% 20–29 years, 12.32% were 30–39 years, 15.46% were 40–49 years, 17.66 were 50–59 years, 16.33 % were 60–69 years and 15.85% were above 70 years. Slightly more than half of study sample were white (54.76%) and non-Hispanic (53.08%). With respect to education, 19.63% of study sample had graduate or post-graduate degree, 15.52% had general education or college, 64.16% had high school or below and remaining unknown (0.74%). Majority had transportation (99.40%) and 11.50% lived with family. Significant sex-differences were found in demographic and social characteristics of patients tested for COVID-19, 2020–2021 (*p* < 0.05). Males (vs. females) had significantly higher proportion in the 60–69-year-old interval (17.35 vs. 15.60%) and > 70-years (17.04 vs. 15.01%), and predominantly white (56.10 vs. 53.81%, χ^2^ = 132.2041, *p* < 0.0001). Consequently, among males (vs. females) there was a higher percentage of individuals of Hispanic ethnicity (23.03 vs. 22.17%, χ^2^ = 9.5205, *p* < 0.0001). Slightly higher percentage of male patients had better education level with graduate or post-graduate degree (20.23 vs. 19.31%, χ^2^ = 22.9419, *p* < 0.0001).

**Table 1:** Demographic and social characteristics differences between male and female individuals tested for SARS-CoV-2, 2020–2021 (*N* = 62,310).

	**Total**	**Female**	**Male**	**Significance**	**Simple logistic regression**	**Multiple logistic regression**
**Demographic and social characteristics**, ***n*** **(%)**					**Orc (95% CI)**	**ORa (95% CI)**
**Age**				**χ^2^ = 505.0737, *p* < 0.0001**		
<20 years	8,099 (13.0)	4,160 (11.40)	3,939 (15.26)		1.0	1.0
20–29	5,852 (9.39)	3,756 (10.29)	2,096 (8.12)		**0.59 (0.55, 0.63)**	0.22 (0.006, 8.11)
30–39	7,674 (12.32)	4,991 (13.68)	2,683 (10.40)		**0.57 (0.53, 0.61)**	1.17 (0.05, 26.33)
40–49	9,629 (15.46)	6,001 (16.44)	3,628 (14.06)		**0.64 (0.60, 0.68)**	2.07 (0.09, 49.32)
50–59	11,003 (17.66)	6,418 (17.59)	4,585 (17.77)		**0.75 (0.71, 0.80)**	1.15 (0.05, 25.90)
60–69	10,171 (16.33)	5,694 (15.60)	4,477 (17.35)		**0.83 (0.78, 0.88)**	3.51 (0.15, 81.68)
>70	9,875 (15.85)	5,477 (15.01)	4,398 (17.04)		**0.85 (0.80, 0.90)**	3.00 (0.11, 79.10)
**Race**				**χ^2^ = 132.2041, *p* < 0.0001**		
Black or African American	6,389 (10.30)	4,162 (11.46)	2,224 (8.66)		1.0	1.0
Asian	618 (1.00)	342 (0.94)	276 (1.07)		**1.51 (1.28, 1.78)**	–
White	33,947 (54.76)	19,541 (53.81)	14,406 (56.10)		**1.40 (1.31, 1.46)**	2.19 (0.25, 18.91)
Unknown	21,038 (33.94)	12,267 (33.78)	8,771 (34.16)		**1.34 (1.26, 1.42)**	1.05 (0.13, 8.70)
**Ethnicity**				**χ^2^ = 9.5205, *p* < 0.0001**		
Not Hispanic or Latino	32,492 (53.08)	19,214 (53.57)	13,278 (52.37)		1.0	1.0
Hispanic or Latino	13,791 (22.53)	7,953 (22.17)	5,838 (23.03)		**1.06 (1.02, 1.11)**	0.35 (0.09, 1.38)
Unknown	14,936 (24.40)	8,700 (24.26)	6,236 (24.60)		0.98 (0.93, 1.02)	0.66 (0.23, 1.89)
**Education**				**χ^2^ = 22.9419, *p* < 0.0001**		
Graduate/post-graduate degree	640 (19.63)	412 (19.31)	228 (20.23)		1.0	1.0
High school or below	2,091 (64.13)	1,334 (62.51)	757 (67.17)		1.03 (0.85, 1.23)	0.57 (0.06, 5.79)
General Education/college	506 (15.52)	376(17.62)	130 (11.54)		**0.62 (0.48, 0.81)**	0.12 (0.01, 1.40)
Unknown	24 (0.74)	12 (0.56)	12 (1.06)		1.81 (0.80, 4.10)	–
Transportation				χ^2^ = 0.0522, *p* = 0.819		
No	4 (0.60)	2 (0.54)	2 (0.68)		1.0	
Yes	659 (99.40)	367 (99.46)	292 (99.32)		0.80 (0.11, 5.68)	
**Living arrangement**				χ^2^ = 6.1572, *p* = 0.409		
Alone	510 (3.8)	294 (3.74)	216 (3.88)		1.0	
Family	1,544 (11.50)	915 (11.63)	629 (11.31)		0.94 (0.76, 1.15)	
Institution	23 (0.17)	14 (0.18)	9 (0.16)		0.88 (0.37, 2.06)	
Friend/roommate	103 (0.77)	65 (0.83)	38 (0.68)		0.79 (0.51, 1.23)	
Relative	57 (0.42)	26 (0.33)	31 (0.56)		1.62 (0.94, 2.81)	
Spouse	931 (6.93)	558 (7.09)	373 (6.71)		0.91 (0.73, 1.13)	
Unknown	10,258 (76.40)	5,993 (76.20)	4,265 (76.69)		0.97 (0.81, 1.16)	

Shown in bold are those that are statistically significant at *p* < 0.05.

As shown in Table 2, most of COVID-19 patients had normal oxygen saturation (91.98%) and 8.01% with hypoxemia. In terms of vaccination, few patients had Moderna, US, Inc. (4.34%), Pfizer-BioNTech (2.82%), or other vaccines (e.g., AstraZeneca Pharmaceuticals LP, Novavax, Janssen Products, LP.) (1.29%). Majority of the study sample (90.33%) had routine vaccinations (e.g., Hepatitis B, Human Papilloma Virus (HPV), Influenza, Measles, mumps, and Rubella etc.) (Table 2). With regards to comorbidities, 76.53% of COVID-19 patients in the present study were caffeine users, 36.26% do not exercise, 25.67% were alcohol users, 9.98% were smokers, and 5.72% were drug users. More than half of study participants were obese (54.62%), 27.96% were overweight and 15.87% had normal weight. Significant sex-differences were found in laboratory, vaccination, and comorbidities of individuals tested for COVID-19 (*p* < 0.05, Table 2). Slightly greater proportion of male patients had mild hypoxemia (9.31 vs. 7.12%, χ^2^ = 42.9096, *p* < 0.0001). In terms of risk factors, a higher proportion of males were smokers (11.04 vs. 9.24%, *p* < 0.0001), caffeine users (77.44 vs. 75.97%, *p* = 0.045), alcohol users (30.72 vs. 22.36%, *p* < 0.0001) and drug users (6.60 vs. 5.17%, *p* < 0.0001) compared to females. A higher percentage of females had normal weight (12.48 vs. 18.14%) whereas a higher percentage of male patients were overweight (30.96 vs. 25.95%) or obese (55.13 vs. 54.29%, χ^2^ = 298.4379, *p* < 0.0001). No significant sex-differences were obtained in transportation (*p* = 0.819), living arrangement (*p* = 0.409), exercise (*p* = 0.814), vaccine (*p* = 0.334).

**Table 2:** Laboratory, vaccination, and comorbidities differences between male and female individuals tested for SARS-CoV-2, 2020–2021 (*N* = 62,310).

	**Total**	**Female**	**Male**	**Significance**	**Simple logistic regression**	**Multiple logistic regression**
**Laboratory, vaccination, and comorbidities**, ***n*** **(%)**					**ORc (95% CI)**	**ORa (95% CI)**
**Laboratory**						
**Oxygen saturation**				**χ^2^ = 42.9096, *p* < 0.0001**		
Normal	23,433 (91.98)	13,936 (92.88)	9,497 (90.70)		1.0	1.0
Hypoxemia (mild, moderate, severe)	2,043 (8.01)	1,069 (7.12)	974 (9.31)		**1.32 (1.21, 1.45)**	2.21 (0.32, 4.50)
**Vaccination**						
**CVX_code**				χ^2^ = 4.5764, *p* = 0.334		
Other vaccines (e.g., AstraZeneca Pharmaceuticals LP, Novavax, Janssen Products, LP.)	700 (1.29)	389 (1.22)	314 (1.39)		1.0	
Moderna, US, Inc.	2,359 (4.34)	1,370 (4.33)	989 (4.36)		0.89 (0.75, 1.05)	
Pfizer-BioNTech	1,532 (2.82)	879 (2.78)	653 (2.88)		0.91 (0.76, 1.09)	
Routine vaccinations (e.g., Hepatitis A, Hepatitis B, Influenza, etc.)	49,078 (90.33)	28,633 (90.42)	20,445 (90.21)		0.88 (0.76, 1.02)	
Unknown	660 (1.21)	398 (1.26)	262 (1.16)		0.81 (0.65, 1.004)	
**Comorbidities/risk factors/*****pre-*****existing conditions**, ***n*** **(%)**						
**Smoking status**				**χ^2^ = 123.3616, *p* < 0.0001**		
Non-smoker	17,174 (38.56)	10,639 (40.58)	6,535 (35.66)		1.0	1.0
Smoker	4,445 (9.98)	2,422 (9.24)	2,023 (11.04)		**1.36 (1.27, 1.45)**	1.44 (0.46, 4.54)
Unknown	22,923 (51.46)	13,154 (50.18)	9,769 (53.30)		**1.21 (1.16, 1.26)**	2.88 (0.70, 11.88)
**Body mass index (BMI) status**				**χ^2^ = 298.4379, *P* < 0.0001**		
Normal	6,846 (15.87)	4,684 (18.14)	2,162 (12.48)		**1**.0	1.0
Overweight	12,066 (27.96)	6,703 (25.95)	5,363 (30.96)		**1.70 (1.60, 1.80)**	1.55 (0.34, 7.08)
Obese	23,570 (54.62)	14,021 (54.29)	9,549 (55.13)		**1.44 (1.37, 1.52)**	0.63 (0.16, 2.53)
**Caffeine user**				**χ^2^ = 4.0035, *p* = 0.045**		
No	3,271 (23.47)	2,066 (24.03)	1,205 (22.56)		1.0	
Yes	10,667 (76.53)	6,530 (75.97)	4,137 (77.44)		1.09 (1.00, 1.18)	
**Drug user**				**χ^2^ = 16.8293, *p* < 0.0001**		
No	17,723 (94.28)	10,970 (94.83)	6,753 (93.40)		1.0	1.0
Yes	1,075 (5.72)	598 (5.17)	477 (6.60)		**1.30 (1.14, 1.47)**	1.30 (0.27, 66.23)
**Alcohol user**				**χ^2^ = 255.8570, *p* < 0.0001**		
No	21,694 (74.33)	13,676 (77.64)	8,018 (69.28)		1.0	1.0
Yes	7,493 (25.67)	3,938 (22.36)	3,555 (30.72)		**1.54 (1.46, 1.62)**	**4.89 (1.81, 13.23)**
**Exercise**				χ^2^ = 1.7636, *p* = 0.814		
No	487 (36.26)	317 (37.60)	170 (34.00)		1.0	
Yes	856 (63.74)	526 (62.40)	330 (66.00)		1.17 (0.93, 1.58)	

Shown in bold are those that are statistically significant at *p* < 0.05.

In terms of primary reason for visit, and according to the 10th revision of the International Classification of Disease (ICD-10) (Table 3), 14.29% were primary diagnosed for factors affecting health status and contact with health services, such as individuals confronting health services for examinations, genetic susceptibility to disease) (*n* = 3,972), 9.34% for abnormal clinical and lab findings (*n* = 2,597), 7.41% for diabetes mellitus (*n* = 2,059), 5.23% for metabolic disorders (*n* = 1,453), 4.99% for COVID-19 (1,388), 4.16% for Hypertensive diseases (*n* = 1,156), 3.97% for certain infectious and parasitic diseases (e.g., HIV, TB, etc.) (*n* = 1,103), 3.61% for diseases of thyroid gland (*n* = 1,004), 2.50% for anxiety, associative, stress-related and other nonpsychotic mental disorders (*n* = 696), 2.47% for overweight, obesity and other hyperalimentation (*n* = 687), 2.29% for diseases of the blood and blood-forming organs and other conditions encompassing the immune system (*n* = 637), 1.46% for injury, poisoning and other external causes (*n* = 405), 0.85% for mental disorders (e.g., disorders of adult personality and behavior, intellectual disabilities; *n* = 237), and 0.76% for influenza and pneumonia (*n* = 212).

**Table 3:** Primary reason for visit [international classification of diseases 10th revision (ICD-10)] differences between male and female individuals tested for SARS-CoV-2, 2020–2021 (*N* = 62,310).

	**Total**	**Female**	**Male**	**Significance**	**Simple logistic regression**	**Multiple logistic regression**
					**ORc (95% CI)**	**ORa (95% CI)**
**Primary reason for visit (ICD10)**				**χ^2^ = 600.9711, *p* = 0.017**		
**COVID-19**	1,388 (4.99)	775 (4.69)	613 (5.44)		**2.65 (2.16, 3.25)**	2.23 (0.06, 86.36)
**Abnormal clinical and lab findings**	2,597 (9.34)	1,501 (9.08)	1,096 (9.73)		**2.45 (2.02, 2.96)**	**13.82 (1.19, 159.92)**
**Mental, Behavioral and Neurodevelopmental disorders**						
Anxiety, dissociative, stress-related, and other nonpsychotic mental disorders	696 (2.50)	477 (2.88)	219 (1.94)		**1.54 (1.21, 1.95)**	5.51 (0.03, 902.75)
Mental disorders (e.g., disorders of adult personality and behavior, intellectual disabilities)	237 (0.85)	112 (0.68)	125 (1.11)		**3.74 (2.74, 5.09)**	**89.72 (3.34, 2,413.21)**
**Diseases of the blood and blood-forming organs and certain disorders involving the immune mechanism**	637 (2.29)	432 (2.61)	205 (1.82)		**1.59 (1.25, 2.02)**	4.42 (0.29, 68.07)
**Diseases of the circulatory system**						
Hypertensive diseases	1,156 (4.16)	660 (3.99)	496 (4.40)		**2.52 (2.04, 3.11)**	**22.90 (2.17, 241.09)**
**Endocrine**						
Diabetes Mellitus	2,059 (7.41)	1,066 (6.45)	993 (8.82)		**3.12 (2.57, 3.79)**	**19.97 (1.96, 203.84)**
Metabolic disorders	1,453 (5.23)	716 (4.33)	737 (6.54)		**3.45 (2.82, 4.22)**	4.65 (0.29, 73.54)
Disorders of thyroid gland	1,004 (3.61)	723 (4.37)	281 (2.49)		**1.30 (1.04, 1.63)**	21.40 (0.81, 2,871,079)
Overweight, obesity and other hyperalimentation	687 (2.47)	409 (2.47)	409 (2.47)		**2.28 (1.80, 2.87)**	23.51 (1.26, 439.98)
**Certain infectious and parasitic diseases (e.g., HIV, TB, etc.)**	1,103 (3.97)	674 (4.08)	429 (3.81)		0.94 (0.84, 1.05)	1.12 (0.05, 26.75)
**Diseases of the respiratory system**						
Influenza and pneumonia	212 (0.76)	108 (0.65)	104 (0.92)		**1.16 (0.91, 1.40)**	**66.19 (1.02, 4,288.9)**
**Factors influencing health status and contact with health services** (e.g., persons encountering health services for examinations, genetic susceptibility to disease)	3,972 (14.29)	2,412 (14.58)	1,560 (13.85)		**2.17 (1.80, 2.61)**	3.55 (0, 36.9734)
**Injury, poisoning, and other external causes**	405 (1.46)	231 (1.40)	174 (1.54)		**2.52 (1.94, 3.28)**	

Shown in bold are those that are statistically significant at *p* < 0.05.

Significant sex-differences were found in primary reason for visit (ICD-10) of individuals tested for SARS-CoV-2 (*p* < 0.05, Table 3). A higher proportion of male patients had abnormal clinical and lab findings (9.73 vs. 9.08%), hypertensive diseases (4.40 vs. 3.99%) and diabetes (8.82 vs. 6.45%) compared to female patients (χ^2^ = 600.9711, *p* = 0.017, Table 3). Whereas a higher proportion of female patients had factors affecting health status and contact with health services (14.58 vs. 13.85%), diseases of thyroid gland (4.37 vs. 2.49%) in addition to anxiety, dissociative, stress-related, and other nonpsychotic mental disorders (2.88 vs. 1.94%) (*p* = 0.017).

Simple logistic regression showed significant sex-differences for age, race, ethnicity, education, laboratory parameters, smoking status, BMI status, caffeine user, drug user, alcohol user, primary reason for visit (except certain infectious and parasitic diseases) (Tables 1–3). For example, a greater proportion of males identifying with Asian race (ORc = 1.51; 95% CI: 1.28, 1.78), White race (ORc = 1.40; 95% CI: 1.31, 1.46) and Hispanic or Latino ethnicity (ORc = 1.06; 95% CI: 1.02, 1.11) compared to females (Table 1). As compared to females, a lower proportion of males had general or college education (ORc = 0.62; 95% CI: 0.48, 0.81). Hypoxemia was 32% more likely among male patients in comparison to female patients (ORc = 1.32; 95% CI: 1.21, 1.45, see Table 2). Male patients had a significantly higher likelihood of smoking as compared to females (ORc = 1.36; 95% CI: 1.27, 1.45). Male COVID-19 patients were 40% more likely to be obese and 70% more likely to be overweight compared to females. In addition, males had significantly higher risk of drug and alcohol use (ORc = 1.30; 95% CI: 1.14, 1.47 and ORc = 1.54; 95% CI: 1.46, 1.62, Table 2). Furthermore, males had significantly higher odds of diseases and related health problems such as abnormal clinical and lab findings (ORc = 2.45; 95% CI: 2.02, 2.96), hypertensive diseases (ORc = 2.52; 95% CI: 2.04, 3.11) and metabolic disorders (ORc = 3.45; 95% CI: 2.82, 4.22) (Table 3).

Findings from multiple logistic regression showed sex-differences in COVID-19 for alcohol use and primary reason for visit (ICD10). Abnormal clinical and lab findings were significantly more frequent in males (ORa = 13.82; 95% CI: 1.19, 159.92) (Table 3). Influenza and pneumonia were more likely among male patients in comparison to female patients (ORa = 66.19; 95% CI: 1.02, 288.9). Men significantly suffered more from mental disorders (e.g., disorders of adult personality and behavior, intellectual disabilities) than women (ORa = 89.72; 95% CI: 3.34, 24,113.21, Table 3). Male COVID-19 patients showed high frequency of underlying comorbidities including hypertensive diseases (ORa = 22.90; 95% CI: 2.17, 241.09) and diabetes (ORa = 66.19; 95% CI: 1.02, 4,228.9), even after adjusting for significant covariates such as age, education and ethnicity.

## Discussion

Using a large US-based cohort, we have observed important sex-dependent disparities in risk factors and disease comorbidities associated with COVID-19. In particular, male patients showed high frequency of underlying comorbidities including abnormal clinical and lab findings, hypertensive diseases, diabetes, whilst adjusting for significant covariates such as age, education and ethnicity.

The results in the present study are in line with other COVID-19 studies conducted globally including US, Europe and China, all which showed that men and women are disproportionally affected. Initial data revealed that males tend to suffer from more severe disease than females, resulting in higher mortality of males vs. females (19–21). Findings from a US-based cohort study of male and female patients revealed a strong independent relationship between male sex and higher COVID-19 susceptibility, bigger chance of ICU admission, use of mechanical ventilation along with longer length of stay—all clinical signs for higher severity of the COVID-19 disease (22). According to a recent meta-analysis of 229 case studies involving more than 10 million individuals, men were found to have a higher risk of contracting COVID-19 than women, and when contracted, they tended to have a higher risk of hospitalization, a higher risk of developing severe COVID-19, a higher need for intensive care, and a higher risk of dying from the infection (23). Another study in China showed that while males and females had equal prevalence of COVID-19, men were 2.4 times more prone to death (24). On the other hand, some evidence showed that women had higher infection risks than males; at older ages, the converse is true (25). Of note, the higher contact intensity of women and their employment in healthcare professions may have contributed to a higher rate of PCR tests being performed and a consequent decrease in the number of undiagnosed cases, which may explain the gender-specific diagnosis in favor of women. Women are also more concerned about their health than males are. Despite the general scarcity of information regarding COVID-19, there exist some gender differences in the search for health information, with females surpassing males. Additionally, men frequently underestimate their health risks, which in turn may lead them to ignore health education messaging (25).

In our study population, a greater proportion of male COVID-19 patients were alcohol users and had multiple comorbidities such as diabetes, hypertension, and metabolic disorders in the adjusted model. The existence of comorbidities tends to increase the risk of adverse COVID-19 outcomes, and more men than women have the usual comorbidities of COVID-19. For instance, hypertension is frequently mentioned as the most prevalent comorbidity in hospitalized COVID-19 patients, and initial data indicated that males had higher levels of hypertension than females for those below 65 years of age (26).

Sex disparities in severity and mortality were also attributed to a higher rate of risky-behaviors and higher existence of comorbidities (i.e., cardiovascular disease, diabetes, etc.) in males than females (4, 19). For example, males are more involved in a lot of risky-behaviors, like smoking and alcohol consumption (4, 19, 27). Smoking has also been associated to adverse COVID-19 outcomes. As an example, smokers were 1.4 times more likely to experience severe COVID-19 symptoms than non-smokers (5, 28). The possible causes include *systemic problems* (mostly cardiovascular) that are more frequent in smokers than non-smokers. Smoking has been linked to higher COVID-19 severity, as well as premature cardiovascular disease and chronic obstructive pulmonary disease (29). *Innate immune cells*, such as the respiratory epithelium, macrophages and lymphocytes, are suppressed by tobacco smoke. Tobacco contains elements that interfere with the respiratory system's natural epithelial lining, increasing oxidative damage and impairing mucociliary clearance. Smokers may be more prone to *pneumonia* since smoking also reduces the ability of the body to produce surfactant, which impacts host immunity and leucocyte performance. Smoking also has a considerable negative impact on *alveolar macrophage activity*, which results in less efficient removal of debris and inflammatory cells from the lungs. Additionally, smoking can change *T-cell reactions*, which can increase vulnerability to respiratory tract infections. This can be specifically harmful for people who already have COVID-19 (30). Nevertheless, probable biologic mechanisms by which smoking may be protective in COVID-19 contain an *anti-inflammatory* effect of nicotine, a *blunted immune response* in smokers and increased nitric oxide in the respiratory tract.

Further, emerging evidence showed that smoking tend to increase the expression of the COVID-19 receptor, ACE2, in the lungs, which could explain why this subset of patients has a higher prevalence of COVID-19 (31). Trends from the most affected countries including US, Italy and China, revealed that males smoke more than females (27, 32). Additionally, this trend is also shown globally, which may further support for the gender disparities in COVID-19 outcomes. Additionally, the aforementioned behavioral factors, like smoking and alcohol intake, predispose men to comorbid conditions including respiratory condition, hypertension and cardiovascular disease, all of which are risk factors for dying (33). This could also explain why men have a greater overall death rate (27, 34, 35).

Social gender roles and sex differences are linked and both have an impact on the incidence and outcomes of the COVID-19. Even during the containment period, males are frequently employed in basic industries and professions that demand them to be active and engaged in social interactions (e.g., food or pharmacy manufacturing and sales, agriculture, transportation, security, etc…). As a results, the majority of men leave their homes and go out with other people, drinking and smoking while taking off their masks. This in turn leads to a higher risk for infection with COVID-19. In the US for instance, men account for most agricultural workers (76%) and for construction, maintenance, and repair workforce (96%) (36), whereas US women tend to hold more administrative, secretarial, and teaching jobs all of which were switched remotely during the pandemic. However, women are more likely to perform paid/unpaid domestic and caregiving roles which also leads to a high risk of contracting COVID-19 (36, 37). Research studies showed that women and girls are more likely to report using masks, washing their hands, and following other public health and social distancing advice (3, 38). Additionally, there are many social norms that demotivate men from obtaining medical care or consulting a doctor, which in turn could increase the likelihood of negative outcomes following infection with COVID-19 (26).

The severity of COVID-19 may also be influenced by additional biological mechanisms of male sex bias, notably with regard to immunological responses. Additionally, it is well-known that men and women react to self-antigens and foreign antigens differently, and gender disparities in the immune response are well-established (39, 40). The fact that male patients had greater plasma levels of innate immune cytokines including IL-8 and IL-18 as well as more robust activation of non-classical monocytes could be possible justification for the actual sex biases (39, 40). Contrarily, during COVID-19 infection, female patients had more robust T cell activation (40). Of note, research studies have shown that a poor T cell response was associated with worse disease outcome in males, and that this association was negatively correlated with patients' age (39). Further studies have demonstrated that estrogen increases endothelial nitric oxide synthase transcriptional activity, which in turn increases nitric oxide (NO) production (41). Females typically experience less serious COVID-19 infection outcomes, which may be related to the effect of estrogen on NO in females, as well as the function of NO as a virus replication inhibitor (42). Emerging evidence has found that some comorbidities, such as obesity and obstructive sleep apnea, may reduce plasma level of testosterone and these comorbidities are common in COVID-19 patients (43, 44). Here, the higher cases observed in male vs. female patients may also be due to greater number of male patients being diabetic, obese and had hypertension, especially older age males (>50 years).

Age could also partially explain the stark differences in risk of COVID-19 reported in the present study. Males (vs. females) patients in our study sample were significantly older with higher proportion aged 60–69, and >70-years. Previously, it was found that mortality and fatality rates, which increase with age, are paramount in men over 50 years old (5, 15). Most COVID-19 deaths occurred in patients over the age of 50, and the sex-dependent risk of poor outcomes increased with age. In addition, the risk of mortality was also higher in patients over the age of 50 in comparison to an equaled group of females of same age (19).

On the contrary, only few studies showed that female patients were at a higher risk for generating long term post-COVID symptoms, such as anxiety, depression, or poor sleep quality, than male patients (45, 46). Other factors like increased psychological stress could also trigger the generation of post-COVID symptoms. Previous studies revealed that COVID-19 pandemic surrounding factors like sleep deprivation, isolation and stress, could also be a risk for generating more post-COVID symptoms in female patients.

### Strength and limitations

This is the first study to examine sex-differences in COVID-19, underlying risk factors and health conditions in a large and consistent sample covering U.S. population. The main strengths of our proposed study include big data approach and straight access to empirical evidence. Additionally, our methodology ensures that there is no bias in patient screening process. However, there are some limitations to our present study. Electronic health record (EHR) may be subject to a possible bias in data recording due to variations between EHR system functionalities and lay-out, coding systems, knowledge and education of the use of EHR system, data extraction tools and data processing. In our study, the number of diagnosed female patients are higher compared to males while the comorbid conditions that increase severity of disease and complications are higher among males compared to females. The gender-specific diagnosis in favor of females may be explained by the higher contact intensity of women and their employment in health-care professions, which could have contributed to a higher proportion of PCR tests being performed and a consequent increase in the number of diagnosed cases. Case determination relies on the sensitivity and specificity of the used PCR testing; a little percentage of people who underwent several tests may have been incorrectly diagnosed in the first encounter. Our study also looked at the comorbidities that might contribute to the observed sex disparities. Even though there is compelling evidence supporting the importance of biological pathways, further research is still needed to investigate how socio-behavioral factors might affect health outcomes.

## Conclusion

In conclusion, sex-based differences exist in high-risk behavior and comorbidities among a large, US-based cohort. In advanced age, the gender-specific risk is mainly more noticeable. According to this study, sex should be given more consideration when interpreting COVID-19 data. Clinicians will be able to make suitable patient-tailored medical decisions with the use of sex-disaggregated data. Understanding the differences in outcomes between male and female patients will inform gender equity responsive approach to COVID-19 outbreak and enhance the effectiveness of clinical practice, health policy and interventions. Future research is required to understand the causes of the gender difference and may also be of potential interest for public health decision-makers.

## Data availability statement

The data analyzed in this study is subject to the following licenses/restrictions: The data that support the findings of this study are available from Healthjump database and the COVID-19 Research Database consortium but restrictions apply to the availability of these data, which were used under license for the current study, and so are not publicly available. Requests to access these datasets should be directed to the database and analysis environment used for the study were provided by the COVID-19 Research Database consortium (https://covid19researchdatabase.org).

## Author contributions

SK participated in the conceptualization of the idea, investigation, and project administration. SK and MD contributed to the data curation, formal analysis, methodology, software, validation, visualization, manuscript drafting, and the final review of the manuscript. Both authors have read and approved the final manuscript.

## Funding

The present study is funded by the Health Care Cost Institute, Datavant and the COVID-19 Research Database Grant Accelerator Program, Bill and Melinda Gates Foundation (Project Number CORONAVIRUSHUB-D-21-00081).

## Conflict of interest

The authors declare that the research was conducted in the absence of any commercial or financial relationships that could be construed as a potential conflict of interest.

## Publisher's note

All claims expressed in this article are solely those of the authors and do not necessarily represent those of their affiliated organizations, or those of the publisher, the editors and the reviewers. Any product that may be evaluated in this article, or claim that may be made by its manufacturer, is not guaranteed or endorsed by the publisher.
